# ALTURA™ Stent Graft Shortening and Its Implications After EVAR

**DOI:** 10.3390/jcm14041157

**Published:** 2025-02-11

**Authors:** Artis Knapsis, Melik-Murathan Seker, Markus Udo Wagenhäuser, Julian-Dario Rembe, Janis Savlovskis, Hubert Schelzig, Dainis Krievins, Alexander Oberhuber

**Affiliations:** 1 Department of Vascular and Endovascular Surgery, Medical Faculty and University Hospital Düsseldorf, Heinrich-Heine-University, 40225 Duesseldorf, Germany; melik-murathan.seker@uni-duesseldorf.de (M.-M.S.); markus.wagenhaeuser@med.uni-duesseldorf.de (M.U.W.); julian-dario.rembe@med.uni-duesseldorf.de (J.-D.R.); hubert.schelzig@med.uni-duesseldorf.de (H.S.); 2 Department of Invasive Radiology, Pauls Stradins Clinical University Hospital, LV-1002 Riga, Latvia; janis.savlovskis@gmail.com; 3 Department of Vascular Surgery, Pauls Stradins Clinical University Hospital, LV-1002 Riga, Latvia; dainis.krievins@gmail.com; 4 Department of Vascular and Endovascular Surgery, University Hospital Münster, 48149 Münster, Germany; alexander.oberhuber@ukmuenster.de

**Keywords:** endovascular aortic repair, stent graft migration, ALTURA™ stent graft system, stent graft shortening, endoleak, aneurysm sack shrinkage

## Abstract

**Objectives:** The ALTURA™ stent graft system is designed for the treatment of abdominal aortic and/or aorto-iliac aneurysms. This study evaluates the performance of the ALTURA™ stent graft, focusing on AAA diameter, landing zones, stent graft length, and migration following endovascular aortic repair (EVAR). **Methods:** This is a retrospective analysis of computed tomography (CT) images focuses on patients with infrarenal abdominal aortic aneurysm (AAA) treated with the ALTURA™ stent graft system (Lombard, Ltd., Didcot, UK) at Pauls Stradins Clinical University Hospital in Riga, Latvia, and University Hospital Düsseldorf in Düsseldorf, Germany. The study population consisted of patients with asymptomatic AAAs who underwent elective treatment between January 2014 and June 2017. Follow-up CT scans were performed at one month, six months, one, two, and three years after implantation. Changes in stent graft length, aneurysm sac diameter, and the proximal and distal sealing zones were evaluated. **Results:** A retrospective analysis was conducted on computed tomography (CT) images from 40 patients (mean age 70.4 ± 8.5 years, 34 males, 6 females) who were treated with the ALTURA™ stent graft system for infrarenal abdominal aortic aneurysms (mean aneurysm diameter 5.6 ± 1.0 cm). The mean follow-up duration was 24.2 ± 10.6 months, with CT scans completed for all patients at one month and for 80% at one year. The mean total shortening of the stent graft one year after EVAR was 4 ± 3 mm (*p* < 0.001), 7 ± 5 mm after two years (*p* < 0.001), and 9 ± 6 mm after three years (*p* < 0.001). The iliac extensions shortened by 4 ± 3 mm after one year (*p* < 0.001), 6 ± 4 mm after two years (*p* < 0.001), and 8 ± 4 mm after three years (*p* < 0.001). Significant shortening was observed in the iliac extension, while changes in the aortic stent graft were not statistically significant. The reduction in the distal sealing zone and upward migration of the stent graft were 3 ± 3 mm after one year (*p* < 0.001), 5 ± 5 mm after two years (*p* < 0.001), and 7 ± 7 mm after three years (*p* < 0.001). Over the follow-up period, significant stent graft shortening and loss of the distal sealing zone were observed. However, these changes remained within a clinically acceptable range and did not lead to type I endoleak. Aneurysm sac shrinkage greater than 10 mm one year after treatment was observed in 25% of patients (*p* < 0.001). No aneurysm ruptures or AAA-related deaths were reported. **Conclusions:** Significant shortening of ALTURA™ stent graft, migration, and sealing zone reduction were observed without clinical impact after three years. However, in patients with short distal sealing zones, these changes could increase the risk of type Ib endoleak. Longer follow-up is needed to assess long-term durability.

## 1. Introduction

**Figure 1:**
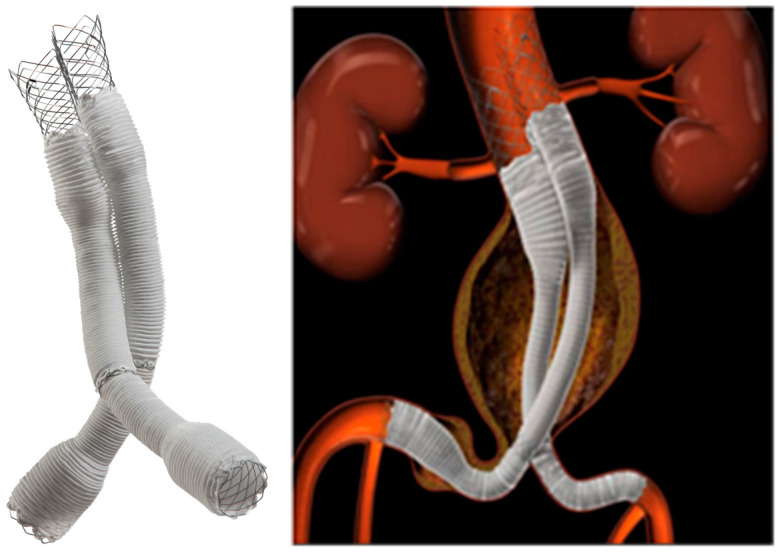
The ALTURA™ stent graft system is uniquely designed with two bilateral ‘D’-shaped proximal aortic stent grafts, complemented by iliac extensions.

Over the past 35 years, stent graft systems for treating abdominal aortic aneurysms (AAAs) have evolved significantly, with most stent graft main bodies now featuring a Y-shaped architecture. However, conventional stent graft systems are often limited by strict anatomical requirements, challenging deployment in patients with short or highly angulated proximal necks, or narrow, tortuous iliac vessels. These limitations can exclude a significant subset of patients from benefiting from EVAR. The ALTURA™ (Lombard, Ltd., Didcot, UK) stent graft system has been designed to address these challenges, offering features such as a lower-profile delivery system and a modular design that enables greater anatomical adaptability. The ALTURA™ stent graft system by Lombard Medical Limited, designed for infrarenal AAAs, is a low-profile (14 French) device with a unique design that includes two bilateral “D”-shaped proximal aortic stent grafts and iliac extensions. It allows precise placement below the renal arteries and above the hypogastric arteries, eliminating the need for contralateral gate cannulation and simplifying the procedure (Figure 1).

**Figure 2:**
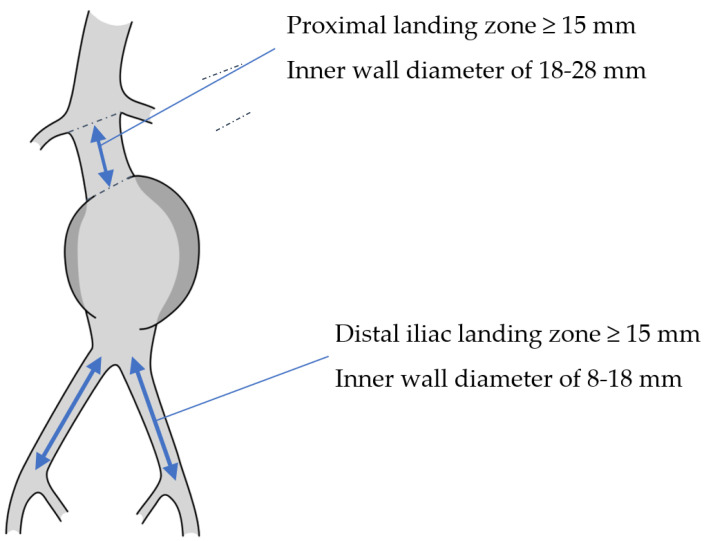
Abdominal aortic aneurysm showing the instructions for use for the ALTURA™ stent graft system.

The ALTURA™ stent graft system is designed for the treatment of abdominal aortic and/or aorto-iliac aneurysms in patients with vascular morphology meeting specific anatomical criteria, including adequate iliac/femoral access compatible with 14 French introducer sheaths, a non-aneurysmal proximal aortic neck with a length of ≥15 mm, an inner wall diameter of 18–28 mm, and a neck angulation of ≤60°, as well as a distal common iliac landing zone with an inner wall diameter of 8–18 mm and a length of ≥15 mm (Figure 2). This study evaluates the performance of the ALTURA™ stent graft, focusing on AAA diameter, landing zones, stent graft length, and migration following endovascular aortic repair (EVAR).

## 2. Materials and Methods

### 2.1. Study Design

This retrospective analysis of computed tomography (CT) images focuses on patients with infrarenal AAA treated with the ALTURA™ stent graft system (Lombard, Ltd., UK) at Pauls Stradins Clinical University Hospital in Riga, Latvia, and University Hospital Düsseldorf in Düsseldorf, Germany. The study protocol was approved by the local ethics committees at both sites (Study No.: PSKUS-3218725 and 6033R, approval date on 17 August 2017 and 21 June 2017, respectively).

### 2.2. Study Population

The study population consisted of patients with AAAs who underwent elective treatment between January 2014 and June 2017. Included were all elective patients over the age of 18 with asymptomatic AAAs who received treatment with the ALTURA™ stent graft system, along with pre-implant, post-implant, and follow-up CT scans. Patients were excluded if they did not meet the inclusion criteria, had symptomatic or ruptured infrarenal AAAs, or had mycotic or systemic disorder-associated infrarenal AAAs. Data were obtained from existing medical records; no further visits or follow-up calls outside of routine medical follow-up were performed. Follow-up CT scans were performed at one month, six months, one, two, and three years after implantation.

### 2.3. Quantitative Morphometric Analysis

**Figure 3:**
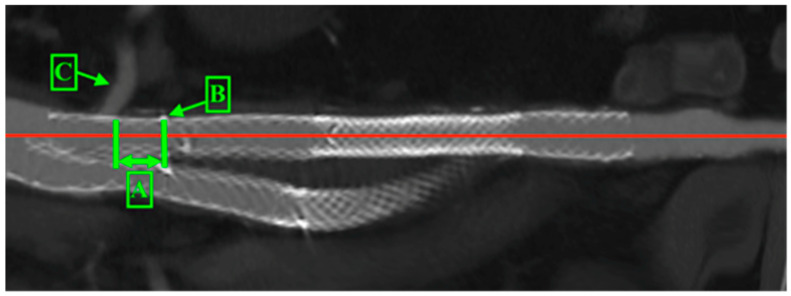
Proximal sealing zone. A—distance from renal artery to renal radiopaque marker, B—renal artery radiopaque marker, C—renal artery. The red line is the central line of the stent graft and artery.

The available CT scans underwent a quantitative morphometric assessment using OsiriX v.5.8.2 (Pixmeo SARL, Bernex, Switzerland). The scans were performed with 256-level scanners, featuring an axial level thickness of 1 mm. All analyses were based on multi-planar aortic reconstructions, with measurements taken perpendicular to the vessel or stent graft centreline. The migration of the stent graft and changes in the proximal landing zone were measured from the lowermost renal artery to the stent graft’s renal artery radiopaque marker (Figure 3).

**Figure 4:**
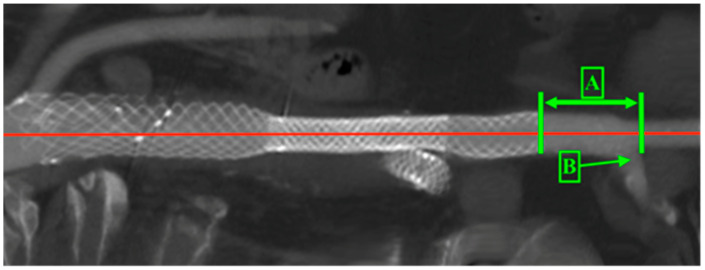
Distal sealing zone. A—distance from end of the stent graft to external iliac artery, B—internal iliac artery. The red line is the central line of the stent graft and artery.

The migration of the stent graft within the iliac arteries and changes in the distal sealing zone were measured from the external iliac artery to the distal end of the stent graft (Figure 4).

**Figure 5:**
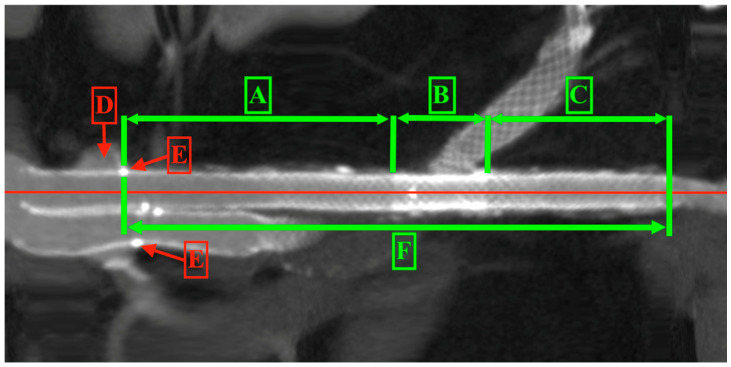
ALTURA™ stent graft parts and segments. A—proximal part of aortic stent graft, B—overlapping zone of aortic stent graft and iliac extension, **A and B—aortic stent graft**, C—distal part of iliac extension, **B and C—iliac extension**, D—renal artery, E—renal artery radiopaque marker, F—total length of stent graft. The red line is the central line of the stent graft and artery.

Measurements included the total length of the stent graft defined as the segments A and B, the length of the proximal part of the D-shaped stent graft, the aortic stent graft, the overlapping zone, the iliac stent graft defined as the segments B and C, and the distal part of the iliac extension (Figure 5).

Migration was defined as either an increase in distance by >10 mm between the renal artery and the proximal marker of the D-shaped graft, or an increase in distance by >10 mm between the external iliac artery and the distal end of the iliac extension.

The maximal aneurysm sac diameter was calculated from the cross-sectional area using a standard equation. In this study only one person collected the data and conducted the measurements, and so interobserver variability was not present.

All data were collected and anonymously entered into an electronic database (Microsoft Excel; Microsoft Corporation, Redmond, WA, USA). Statistical analysis was performed using SPSS 20.0 (SPSS Inc., Chicago, IL, USA). No formal case number or power calculation was performed since, due to the retrospective design, only a specific limited number of patients for the analysis cohort was available within the chosen inclusion timeframe and study centers. All cases fulfilled the inclusion criteria and yielded complete datasets for at least the first follow-up time-point (30 days). Continuous variables are presented as mean ± standard deviation (SD), while categorical variables are presented as absolute number of patients and percentage [*n* (%)]. Nonparametric data were analyzed using the Wilcoxon Test for comparing two groups and the Friedman Test for comparing three or more groups. Kaplan–Meier survival analysis was used to estimate freedom from endoleaks and secondary procedures. A *p*-value of less than 0.05 was considered statistically significant.

## 3. Results

A total of 174 CT scans were analyzed, including 40 performed before EVAR and 134 after EVAR. Each patient had two separate parallel stent grafts, resulting in 268 center lumen line-based reconstructions.

### 3.1. Patient Population

Between January 2014 and June 2017, 40 patients with a mean age of 70.4 ± 8.5 years (range: 47–84 years) were treated with the ALTURA™ stent graft system. Most of the patients were male (34 men, 6 women). All treatments were performed at two clinical sites: University Hospital Düsseldorf (Germany) and Pauls Stradins Clinical University Hospital (Riga, Latvia). The mean AAA diameter was 5.6 ± 1.0 cm (range: 4.4–8.1 cm). None of the patients had symptomatic or ruptured AAAs. Implantations were performed percutaneously in 95% of cases. The mean operating time was 86 ± 26 min (range: 34–210 min), and the average hospital stay was 3 ± 1.5 days (Table 1).

CT scan follow-up was completed for all 40 patients at 1 month, for 80% of patients at 1 year (32 patients), 60% at 2 years (24 patients), and 20% at 3 years (8 patients). No perioperative deaths were observed. The average follow-up was 24.2 ± 10.6 months (Table 2).

### 3.2. Aneurysm Sac Changes

Aneurysm sac shrinkage greater than 10 mm one year after treatment, compared to the pre-treatment diameter, was observed in 25% of patients (Table 3). After two years, aneurysm sac shrinkage greater than 10 mm was seen in 9 of 24 patients (38%).

**Figure 6:**
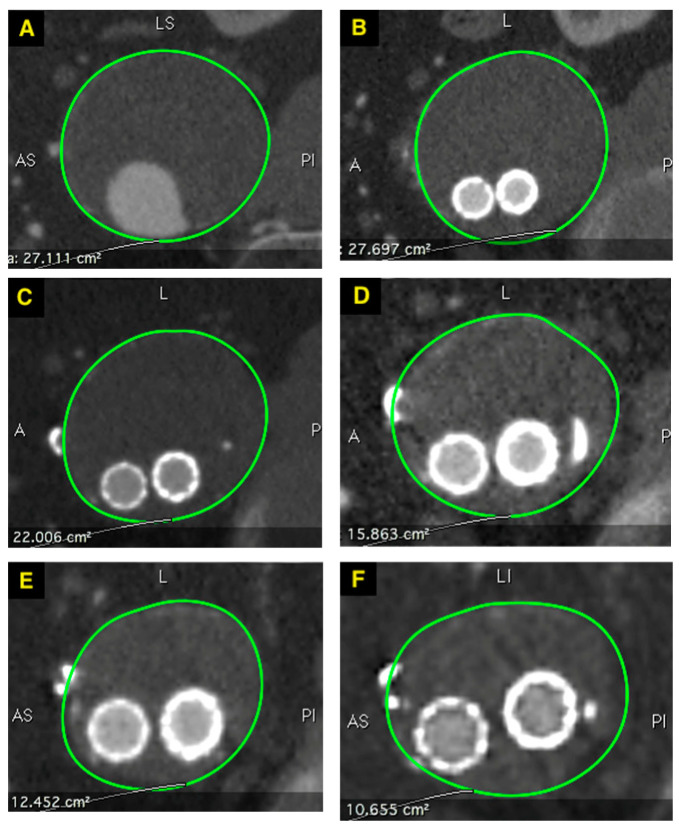
Changes of maximal aneurysm sac cross sectional area over time. (**A**)—before treatment 27.1 cm^2^, (**B**)—1st month follow-up 27.7 cm^2^, (**C**)—6th month follow-up 22.1 cm^2^, (**D**)—1st year follow-up 15.9 cm^2^, (**E**)—2nd year follow-up 12.4 cm^2^, (**F**)—3rd year follow-up 10.7 cm^2^.

The maximum aneurysm sac growth was 6 mm, observed in 12% of patients (3/24), all of whom had a type II endoleak identified at the first-month follow-up. The mean aneurysm sac shrinkage compared to the pre-treatment diameter was 6 ± 7 mm after one year, 7 ± 11 mm after two years, and 10 ± 14 mm after three years (Figure 6). Statistically significant aneurysm sac shrinkage was observed one year after stent graft implantation (*p* < 0.001).

### 3.3. Stent Graft Shortening

**Figure 7:**
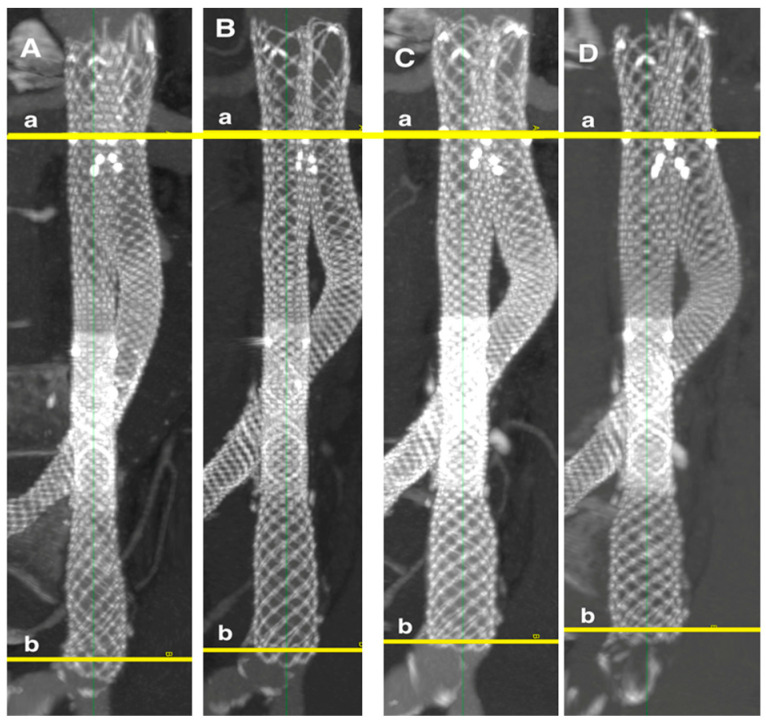
Shortening of Altura stent graft. (**A**)—1st month follow-up with stent graft length 17.3 cm, (**B**)—1st year—16.6 cm, (**C**)—2nd year—15.9 cm (**D**)—3rd year—15.7 cm, a—aortic stent graft renal artery radiopaque marker, b—end of the iliac stent graft.

The mean total stent graft shortening one year after EVAR, compared to the first CT scan follow-up one month after EVAR, was 4 ± 3 mm. The total stent graft shortening two years after EVAR was 7 ± 5 mm, and three years after EVAR was 9 ± 6 mm (Figure 7).

Stent graft shortening greater than 10 mm, compared with the first CT scan follow-up one month after EVAR, was observed in 3% of stent grafts (2/63) after one year, 27% (13/48) after two years, and 50% (8/16) after three years.

The total shortening of the ALTURA™ stent graft system at one, two, and three years after implantation was statistically significant compared to the first follow-up one month after EVAR (*p* < 0.001). Significant shortening was observed in the iliac extension, while changes in the aortic stent graft were not statistically significant (Table 4).

Specifically, aortic stent graft shortening was 1 ± 3 mm after one year, 2 ± 4 mm after two years, and 4 ± 4 mm after three years. The iliac extensions shortened by 4 ± 3 mm after one year, 6 ± 4 mm after two years, and 8 ± 4 mm after three years. The shortening of the overlapping zone and iliac extensions was statistically significant over the three-year period (*p* < 0.002).

### 3.4. Reduction of the Sealing Zone

**Figure 8:**
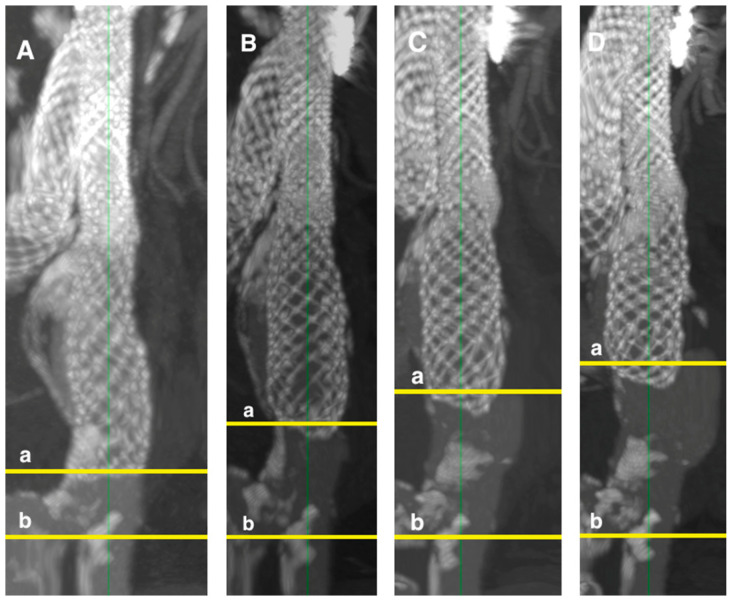
Stent graft upward migration in iliac arteries and reduction of distal sealing zone in patient with aneurysmatic common iliac artery. (**A**)—1st month follow-up, (**B**)—1st year with stent graft upward migration 0.5 cm, (**C**)—2nd year—1.0 cm, (**D**)—3rd year—2.5 cm, a—end of the iliac stent graft, b—external iliac artery. The green line is a central line of stent graft and artery.

The loss of the distal sealing zone and upward migration of the stent graft from the external iliac artery were 3 ± 3 mm after one year, 5 ± 5 mm after two years, and 7 ± 7 mm after three years (Figure 8).

Reduction of the distal sealing zone and upward migration greater than 10 mm was observed in 5% of stent grafts after one year, 26% after two years, and 25% after three years, with statistically significant changes over time (*p* < 0.001) (Figure 8). The most significant upward migration and reduction of the distal sealing zone were seen in patients with aneurysmal common iliac arteries (Table 5).

**Figure 9:**
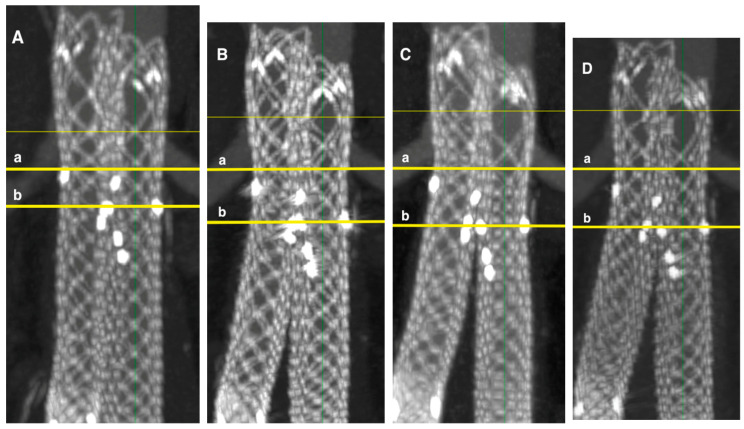
Stent graft downward movement in infrarenal aortic neck and reduction of proximal sealing zone. (**A**)—1st month follow-up, (**B**)—1st year with stent graft downward movement 4 mm, (**C**)—2nd year 5 mm, (**D**)—3rd year 6 mm, a—lowermost renal artery, b—aortic stent graft renal artery radiopaque marker. The green line is a central line of stent graft and artery.

Downward movement and slight reduction in the proximal sealing zone from the lowermost renal artery to the renal artery radiopaque marker were measured at 1 ± 2 mm after one year, 2 ± 2 mm after two years, and 3 ± 3 mm after three years. The maximum downward movement observed across all follow-ups was 7 mm (Figure 9). Although these changes were statistically significant (*p* < 0.001) and demonstrated a trend over time (Table 5), they did not meet the reporting standard for clinically significant migration, which requires a minimum shift of 10 mm. This phenomenon can be explained by the natural behavior of braided stents, which tend to shorten as they expand in diameter.

### 3.5. Secondary Procedures

During the follow-up period, 10 secondary procedures were performed in 8 patients, resulting in a 20% incidence of reintervention. The reasons for these procedures included type I endoleak, type II endoleak with aneurysm sac growth, stent graft stenosis, and limb occlusion. There were no aneurysm-related deaths or ruptures during the study period. Type II endoleak was the leading cause of reintervention (*n* = 6, 60%), followed by stent graft stenosis (*n* = 3, 30%) and stent graft thrombosis (*n* = 1, 10%). Most secondary procedures were catheter-based (*n* = 9, 90%), including embolization (*n* = 6, 67%) and percutaneous transluminal angioplasty (PTA)/stent placement (*n* = 3, 33%). One patient with a type Ia endoleak was treated with coil and glue embolization after the first-month follow-up. Seven patients were diagnosed with type II endoleaks, all identified during the first-month follow-up. Five of these patients underwent glue embolization, with or without coil embolization. Three patients were diagnosed with stent graft stenosis at the first-month follow-up. Two were treated with PTA, while one underwent both PTA and stent implantation. One of the patients with iliac stent stenosis later developed stent graft thrombosis on the same side at the three-year follow-up. This patient required stent graft explantation and open surgical conversion.

## 4. Discussion

The mid-term follow-up results after ALTURA™ stent graft implantation in patients with AAAs reveal both promising outcomes and ongoing challenges associated with EVAR. The aneurysm sac shrinkage observed as early as one-year post-implantation suggests an effective initial endovascular repair of AAA, with further improvements over time, highlighting the durability and efficacy of the stent graft technology in reducing aneurysm-related complications. The observed aneurysm sac shrinkage serves as a key indicator of EVAR success. After one year, 47% of patients exhibited significant aneurysm sac shrinkage (>5 mm), demonstrating the initial effectiveness of the stent graft. Karaolanis et al. reported aneurysm sac shrinkage >3 mm in 35% of patients in their study of the ALTURA™ stent graft, which is consistent with our findings [1]. In addition to the ALTURA™ stent graft, other stent grafts have also been evaluated regarding aneurysm sac shrinkage. Oliveira-Pinto et al. reported that the Medtronic Endurant (Medtronic [Dublin, Ireland]) stent graft resulted in aneurysm sac shrinkage of more than 5 mm in 46.3% of patients. They also reported aneurysm sac shrinkage for the Gore Excluder (W.L. Gore and Associates, Inc., Newark, AZ, USA) in 47.5% of patients, making the results of these stent grafts comparable to ours [2]. However, this does not apply to the Endologix Nellix (Endologix, Inc., Irvine, CA, USA), where Choo et al. reported aneurysm sac enlargement in 3.1% of patients [3]. In addition to aneurysm sac shrinkage, endoleaks are a critical marker for evaluating stent graft performance. In our study, the endoleak rate was consistent with other devices such as the Medtronic Endurant, Artivion E-Tegra, Cook Zenith Alpha, Gore Excluder, and Endologix Nellix [2,3,4,5,6,7,8,9,10,11].

Our primary objective was to assess the morphological changes in the ALTURA™ stent graft system over time. Our results revealed that the stent graft experienced progressive shortening, which was most pronounced in the iliac extension and the overlapping zone between the aortic stent graft and the iliac extension, even as early as one year after implantation. In contrast, the proximal part of the D-shaped aortic stent graft remained relatively stable. The mean total shortening of the stent graft was 4 ± 3 mm after one year, increasing to 7 ± 5 mm after two years, and reaching 9 ± 6 mm after three years. A significant proportion of stent grafts (50%) exhibited shortening greater than 10 mm by the three-year mark.

The shortening was particularly evident in the iliac extensions, which showed a reduction of 4 ± 3 mm at one year, 6 ± 4 mm at two years, and 8 ± 4 mm at three years. In addition to this shortening, there was also notable migration of the stent graft, especially from the distal sealing zone, leading to a reduction of this critical area over time. The upward migration from the external iliac artery was 3 ± 3 mm after one year, increasing to 7 ± 7 mm after three years, with the most significant migration observed in patients with aneurysmal common iliac arteries. This migration resulted in a reduction of the distal sealing zone in a substantial percentage of stent grafts, affecting 25–26% of patients by the two- and three-year follow-ups.

Conversely, downward movement and reduction of the proximal sealing zone from the lowermost renal artery were less pronounced, though still statistically significant, with a maximum observed movement of 7 mm over the three-year period. Despite the significant shortening, migration, and reduction of the distal sealing zone, we found no cases of type Ib endoleak. This may be attributed to the gradual shortening of the stent graft at the distal end over time, or it could reflect the fact that only 20% of patients attended the three-year follow-up, possibly leading to undetected cases. Nevertheless, the risk of developing a type Ib endoleak is higher when the distal portion of the stent graft shortens, which could necessitate reconsideration of the IFU concerning upward migration. An interesting observation is the nature of the shortening that occurs. The results suggest that both the overall stent graft and, in particular, the iliac extension experience more pronounced shortening during the first year. This may be related to the design of the stent graft. The ALTURA™ stent graft is a braided stent graft, which implies a direct correlation between the stent diameter and its length. This could explain why most stent changes occur within the first year.

Due to Altura stent graft shortening and the loss of the distal sealing zone, the distal sealing zone of only 15 mm for the Altura stent graft may be too short and could be associated with a risk of Type Ib endoleak. Studies with longer follow-up are needed.

To our knowledge, very few studies have examined the morphology and changes of the stent graft in as much detail as we have over a three-year period, particularly regarding the distal sealing zone, where data are notably scarce. Roos et al. analyzed factors for reinterventions with additional iliac stent grafts after EVAR. Their study found that most reinterventions requiring additional iliac stent grafts were due to migration, occurring late after primary repair, with a significant proportion caused by rupture. Limbs requiring reinterventions had larger iliac artery diameters, shorter attachment zones, and lower stent graft oversizing at the initial procedure. While most research on EVAR migration focuses on the proximal landing zone, their study also highlights the significant occurrence of distal migration as a common cause of reintervention [12].

We also found that shortening of the distal sealing zone can lead to reinterventions; nevertheless, our comparisons primarily focus on the proximal sealing zone and downward movement, because there is more data to discuss. Oliveira-Pinto et al. reported a movement greater than 5 mm in 4.6% of patients treated with the Medtronic Endurant stent graft, while the rate for the Gore Excluder was 7.6% [2]. Tsolakis et al. also studied the Gore Excluder but combined proximal movement with proximal misdeployment, reporting lower rates of 0.6%. However, Tsolakis et al. also examined distal migration and misdeployment, finding rates of 10.3% for the older version of the Gore Excluder and 2.9% for the newer version [11]. These findings underscore the importance of long-term monitoring of stent grafts, particularly in patients with anatomical variations, such as aneurysmal iliac arteries, as both shortening and migration can compromise the effectiveness of the stent graft system over time.

It is important to know that, like any nitinol aortic stent graft, the ALTURA™ stent graft exerts continuous radial pressure on the sealing zone, leading to gradual dilation of the blood vessel until the graft reaches its nominal diameter. Due to the design of the ALTURA™ stent graft, which features a braided stent construction, there is a direct relationship between the stent’s diameter and its length. This relationship helps explain the primary finding of our study—the observed reduction in the sealing zone over time. The radial force exerted by the graft on the vessel wall leads to gradual expansion, causing the stent graft to shorten. This expansion and contraction process is expected to stabilize once the graft reaches its nominal diameter, meaning that these findings have two important implications, the need for extended follow-up to monitor long-term stability and additional research to investigate the correlation between the degree of graft oversizing at implantation and the extent of stent graft shortening over time. A better understanding of these dynamics could optimize future implantation strategies and improve patient outcomes.

The primary limitation of this study is the small sample size of 40 patients from two sites, with only 20% of patients attending the three-year follow-up. This lower follow-up rate may introduce selection bias, as the individuals remaining in the study could differ systematically from those lost to follow-up. Consequently, the generalizability of findings at later time-points may be affected. Future studies should aim to address this limitation through strategies to improve follow-up rates and assess the potential impact of attrition on study outcomes. Larger cohorts and longer follow-up periods are necessary to fully assess the ALTURA™ stent graft system’s long-term performance.

## 5. Conclusions

Significant shortening and reduction of both the distal and proximal sealing zones of the ALTURA™ stent graft system were observed over time. However, these changes did not result in any clinically significant complications after three years in the studied cohort. The reduction of the distal sealing zone could potentially lead to the development of a type Ib endoleak. Given the shortening of the ALTURA stent graft, a distal sealing zone of only 15 mm in the common iliac artery may not provide sufficient sealing and could increase the risk of a type Ib endoleak. However, given the structural changes over time, longer follow-up studies are strongly recommended to identify the most suitable patients for the ALTURA stent graft system.

## Figures and Tables

**Table 1:** Patient demographics.

Variable	Mean ± SD or *n* (%)
Age (mean ± SD)	70.4 ± 8.5 years
Male gender	34 (85)
Maximum AAA diameter (mean ± SD)	5.6 ± 1.0 cm
Smokers	10 (25)
Diabetes mellitus	5 (13)
Concomitant cardiac disease	24 (60)
Dialysis	0 (0)
Previous cerebral infarction/TIA	2 (5)
Arterial hypertension	30 (75)
Known pulmonary disease	1 (3)
length of stay (mean ± SD)	3 ± 1.5 days
Operation time (mean ± SD)	86 ± 26 min

AAA—abdominal aortic aneurysm; *n*—count; SD—standard deviation; TIA—transient ischemic attack Continuous data are presented as mean ± standard deviation; categorical data are given as counts (percentage).

**Table 2:** Overview of patient follow-up.

	1st Month	1st Year	2nd Year	3rd Year
Patients (*n*)	40	32	24	8
Percentage (%)	100	80	60	20

**Table 3:** Aneurysm sac changes compared with pretreatment aneurysm sac diameter.

Aneurysm Sac	1st Year	2nd Year	3rd Year
Shrinkage in mm	6 ± 7 mm *p* = 0.017	7 ± 11 mm *p* = 0.164	10 ± 14 mm *p* = 0.108
Shrinkage > 5 mm	*n* = 15/32 (47%)	*n* = 13/24 (54%)	*n* = 6/8 (75%)
Shrinkage > 10 mm	*n* = 8/32 (25%)	*n* = 9/24 (38%)	*n* = 4/8 (50%)
Growth > 5 mm	*n* = 1/32 (3%)	*n* = 3/24 (12%)	*n* = 1/8 (12%)

**Table 4:** Shortening of ALTURA stent graft in comparison with first month follow-up.

	Proximal Part of Aortic Stent Graft (mm)	Iliac Extension		Total Stent Graft (mm)
		Overlapping Zone of Aortic Stent Graft and Iliac Extension (mm)	Distal Part of Iliac Extension (mm)	
	Aortic Stent Graft			
1st year	0.2 ± 2 *p* = 0.337	1 ± 2 *p* < 0.001	3 ± 3 *p* < 0.001	4 ± 3 *p* < 0.001
2nd year	0.3 ± 3 *p* = 0.208	2 ± 2 *p* < 0.001	4 ± 3 *p* < 0.001	7 ± 5 *p* < 0.001
3rd year	1.0 ± 4 *p* = 0.264	3 ± 3 *p* = 0.002	4 ± 4 *p* < 0.001	9 ± 6 *p* < 0.001

mm—millimeter; *p*—probability-value; Continuous data are presented as mean ± standard deviation.

**Table 5:** Stent graft downward movement, reduction of proximal and distal sealing zone compared to first month of follow up.

	1st Year	2nd Year	3rd Year
Stent graft downward movement and reduction of proximal sealing zone (mm)	1 ± 2 *p* < 0.001	2 ± 2 *p* < 0.001	3 ± 3 *p* = 0.003
Maximal stent graft downward movement and reduction of proximal sealing zone (mm)	7	7	6
Stent graft upward migration in iliac arteries and reduction of distal sealing zone (mm)	3 ± 3 *p* < 0.001	5 ± 5 *p* < 0.001	7 ± 7 *p* < 0.001
Stent graft upward migration in iliac arteries and reduction of distal sealing zone > 10 mm	5% (3/63)	26% (12/47)	25% (4/16)
Maximal stent graft upward migration in iliac arteries and reduction of distal sealing zone (mm)	15	18	25

*p*—probability-value; Continuous data are presented as mean ± standard deviation; categorical data are given as the percentage (counts).

## Data Availability

The data presented in this study cannot be shared due to the privacy of individuals that participated in this study and to ethical reasons. On justified interest, the data will be available from the corresponding author after approval from the responsible Ethical Committee.

## References

[1] Karaolanis G.I., Hadjis D., Samara E., Gomatos I.P., Tzimas P., Glantzounis G.K. (2023). Low-Profile Altura Endograft System for Endovascular Abdominal Aorta Aneurysm Repair. Preliminary Results in Elective and Emergent Situations. Ann. Vasc. Surg..

[2] Oliveira-Pinto J., Oliveira N.F.G., Bastos-Goncalves F.M., Hoeks S., Rijn M.J.V., Raa S.T., Mansilha A., Verhagen H.J.M. (2020). Long-term results after standard endovascular aneurysm repair with the Endurant and Excluder stent grafts. J. Vasc. Surg..

[3] Choo X.Y., Hajibandeh S., Hajibandeh S., Antoniou G.A. (2019). The Nellix endovascular aneurysm sealing system: Current perspectives. Med. Devices.

[4] Giagtzidis I.T., Konstantinidis K., Kalogirou T.E., Karkos C.D., Papazoglou K.O. (2016). Use of Endurant Stent-Graft Aortic Extensions for the Treatment of Focal Aortic Pathology. Ann. Vasc. Surg..

[5] Omran S., Muller V., Schawe L., Burger M., Kapahnke S., Bruder L., Haidar H., Konietschke F., Greiner A. (2023). Outcomes of Endurant II Stent Graft According to Anatomic Severity Grade Score. J. Endovasc. Ther..

[6] Marone E.M., Rinaldi L.F., Brioschi C., Bracale U.M., Modugno P., Maione M., Curci R., Filippi F., Piffaretti G., Gaggiano A. (2024). Endovascular Aortic Repair With the E-Tegra Device: Preliminary Outcomes From a Multicenter National Registry. J. Endovasc. Ther..

[7] Broda M., Eiberg J., Vogt K., Ohlander J.T., Lawaetz M., Sillesen H., Resch T. (2022). Midterm outcomes of aneurysm repair with the Cook Zenith Alpha abdominal endovascular graft. J. Vasc. Surg..

[8] Carpenter J.P., Cuff R., Buckley C., Healey C., Hussain S., Reijnen M.M., Trani J., Bockler D., Nellix I. (2017). One-year pivotal trial outcomes of the Nellix system for endovascular aneurysm sealing. J. Vasc. Surg..

[9] van den Ham L.H., Holden A., Savlovskis J., Witterbottom A., Ouriel K., Reijnen M., EVAS Type IA Endoleak Study Group (2017). Editor’s Choice—Occurrence and Classification of Proximal Type I Endoleaks After EndoVascular Aneurysm Sealing Using the Nellix Device. Eur. J. Vasc. Endovasc. Surg..

[10] Mortola L., Ferrero E., Quaglino S., Ferri M., Viazzo A., Manzo P., Gaggiano A. (2021). Management of Nellix migration and type Ia endoleak from proximal endovascular aneurysm sealing relining to late open conversion. J. Vasc. Surg..

[11] Tsolakis I.A., Kakkos S.K., Papageorgopoulou C.P., Zampakis P., Kalogeropoulou C., Papadoulas S., Lampropoulos G., Nikolakopoulos K.M., Ntouvas I., Kouri A. (2019). Improved effectiveness of the repositionable GORE EXCLUDER AAA endoprosthesis featuring the C3 delivery system compared with the original GORE EXCLUDER AAA endoprosthesis for within the instructions for use treatment of aortoiliac aneurysms. J. Vasc. Surg..

[12] Roos H., Sandstrom C., Koutouzi G., Jeppsson A., Falkenberg M. (2017). Predisposing Factors for Re-interventions with Additional Iliac Stent Grafts After Endovascular Aortic Repair. Eur. J. Vasc. Endovasc. Surg..

